# Petromurin C Induces Protective Autophagy and Apoptosis in FLT3-ITD-Positive AML: Synergy with Gilteritinib

**DOI:** 10.3390/md18010057

**Published:** 2020-01-16

**Authors:** You Na Ha, Sungmi Song, Barbora Orlikova-Boyer, Claudia Cerella, Christo Christov, Anake Kijjoa, Marc Diederich

**Affiliations:** 1 Department of Pharmacy, College of Pharmacy, Seoul National University, 1 Gwanak-ro, Gwanak-gu, Seoul 08626, Korea; younaha@snu.ac.kr (Y.N.H.); sson35@snu.ac.kr (S.S.); 2 Laboratoire de Biologie Moléculaire du Cancer, Hôpital Kirchberg, 9, rue Edward Steichen, L-2540 Luxembourg, Luxembourg; barbora.orlikova@lbmcc.lu (B.O.-B.); claudia.cerella@lbmcc.lu (C.C.); 3 Service d’Histologie, Faculté de Médicine, Université de Lorraine, *INSERM* U1256 *NGERE*, 54000 Nancy, France; christo.christov@univ-lorraine.fr; 4 *ICBAS*-Instituto de Ciências Biomédicas Abel Salazar, Rua de Jorge Viterbo Ferreira, 228, 4050-313 Porto, Portugal; ankijjoa@icbas.up.pt; 5 Interdisciplinary Centre of Marine and Environmental Research (CIIMAR), Terminal de Cruzeiros do Porto de Lexões, Av. General Norton de Matos s/n, 4450-208 Matosinhos, Portugal

**Keywords:** acute myeloid leukemia, *Aspergillus candidus*, *Epipolasis* sp., cell death, mitochondrial stress, combination treatment

## Abstract

Treatment of acute myeloid leukemia (AML) remains inefficient due to drug resistance and relapse, particularly in patients with FMS-like tyrosine kinase 3 (FLT3)-internal tandem duplication (ITD). Marine-derived natural products have recently been used for drug development against AML. We show in this study that petromurin C, which was isolated from the culture extract of the marine-derived fungus *Aspergillus candidus* KUFA0062, isolated from the marine sponge *Epipolasis* sp., induces early autophagy followed by apoptotic cell death via activation of the intrinsic cell death pathway concomitant with mitochondrial stress and downregulation of Mcl-1 in FLT3-ITD mutated MV4-11 cells. Moreover, petromurin C synergized with the clinically-used FLT3 inhibitor gilteritinib at sub-toxic concentrations. Altogether, our results provide preliminary indications that petromurin C provides anti-leukemic effects alone or in combination with gilteritinib.

## 1. Introduction

Acute Myeloid Leukemia (AML) is a malignant disorder characterized by abnormal growth and differentiation of hematopoietic stem cells (HSCs). Accumulation of immature myeloid precursors in bone marrow and peripheral blood causes development of AML. AML is the most commonly found form of acute leukemia in adults. In addition, AML is strongly dependent on ethnicity showing that the incidence rate of AML in Asia is much lower than that in Western countries [1]. Since overall five-year survival of AML patients is 27.4% [2], novel therapeutic approaches are urgently required.

FMS-like tyrosine kinase 3 (FLT3) is a membrane-bound receptor tyrosine kinase that plays an important role in regulatory processes of hematopoietic cells such as phospholipid metabolism transcription, proliferation, and apoptosis. FLT3-ITD mutations have been found in 15% to 35% of AML patients. FLT3-ITDs showed a strong association with leukocytosis, high blast counts, and normal cytogenetics [3]. Overall survival of patients presenting the FLT3-ITD mutation is dismal.

The development of FLT3-ITD-mutation-targeting drugs has been challenging due to poor bioavailability, insufficient potency, inadequate kinase specificity, and short response to duration [4]. The current status of drugs for treating FLT3-ITD-mutated AML indicates a need for the discovery of novel therapies.

Molecules isolated from marine organisms, especially sponges, have shown great utility as a source of anticancer compounds [5]. *Aspergillus candidus* (Family Aspergillaceae), which is a member of *Aspergillus* section *Candidi*, is not only frequently found as a mold in cereal grains and flour but also in marine organisms. Strains of *A. candidus* produce various secondary metabolites such as chlorflavonin, which is an anti-fungal flavone. Petromurin C, which is a *bis*-indolyl benzenoid, was previously isolated from an extract of *A. candidus* KUFA 0062 cultures, isolated from the marine sponge *Epipolasis* sp., collected from a coral reef at the Similan Island National Park in Phang-Nga province, Southern Thailand. Petromurin C significantly decreased viability of various cancer cell lines representing colorectal, liver, lung, breast, and brain cancer [6]. So far, mechanistic information about cell death induction by petromurin C remains to be provided.

Autophagy is a stress response mechanism that plays a role in removing damaged proteins and organelles and providing energy and metabolic intermediates to sustain homeostasis in cells. However, excessive autophagy can trigger cell death after accumulation of autophagosomes [7]. Apoptosis is characterized by cytoplasmic shrinkage, chromatin condensation, nuclear fragmentation, and, eventually, formation of apoptotic bodies [8]. Whereas the intrinsic pathway triggers a permeabilization of the mitochondrial outer membrane regulated by Bcl-2 (B-cell lymphoma 2) family proteins, which leads to apoptosome formation and pro-caspase-9 activation, initiation of the extrinsic pathway involves death receptor signaling leading to pro-caspase 8 activation [9].

In an effort to develop novel therapeutic approaches against AML, gilteritinib, a dual FLT3, and AXL (from *anexelekto*, “uncontrolled”) inhibitor, was approved as a second generation FLT3 inhibitor by the Food and Drug Administration (FDA) of the USA in November 2018. Gilteritinib treatment led to an overall response rate of 49% in FLT3-mutated patients, compared to 12% in FLT3-wild type patients [10]. Moreover, combination therapies of gilteritinib plus cytarabine (AraC) and idarubicin (IDR) or daunorubicin (DNR) showed complete regression of the tumor compared to gilteritinib alone [11], which underlines the fact that this novel drug might perform best in combination with other pharmacological agents.

Accordingly, we report the antileukemic activity of petromurin C alone and in combination with gilteritinib and explored the related cell stress and cell death mechanisms. We showed that petromurin C reduced viability and proliferation of AML cell lines and induced early formation of protective autophagy in addition to bafilomycin A1, which increased intrinsic cell death compared to petromurin C alone. Furthermore, petromurin C potentialized the cell death-inducing effect of gilteritinib in the FLT3-ITD-mutated AML cell line MV4-11.

## 2. Results

### 2.1. Chemical Structure and Druggability of Petromurin C

**Figure 1:**
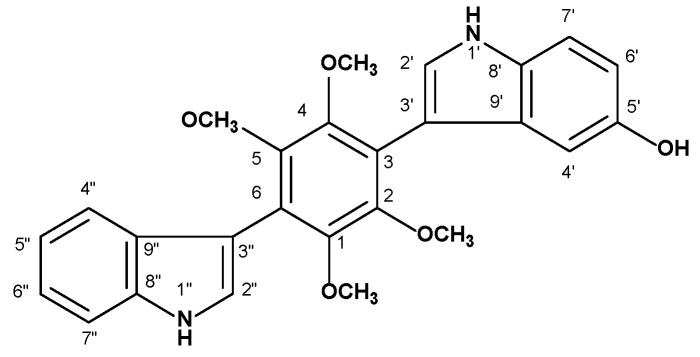
Chemical structure of petromurin C.

To validate the drug-likeness properties of petromurin C (Figure 1), we investigated in silico whether this compound complies with Lipinski’s rule of five. According to this analysis, petromurin C could be suitable for drug development as it has an adequate molecular mass, three hydrogen bond donors, and seven hydrogen bond acceptors, but low lipophilicity (Log*P* > 5) (Table 1) [12].

### 2.2. Cytostatic and Cytotoxic Activities of Petromurin C

**Figure 2:**
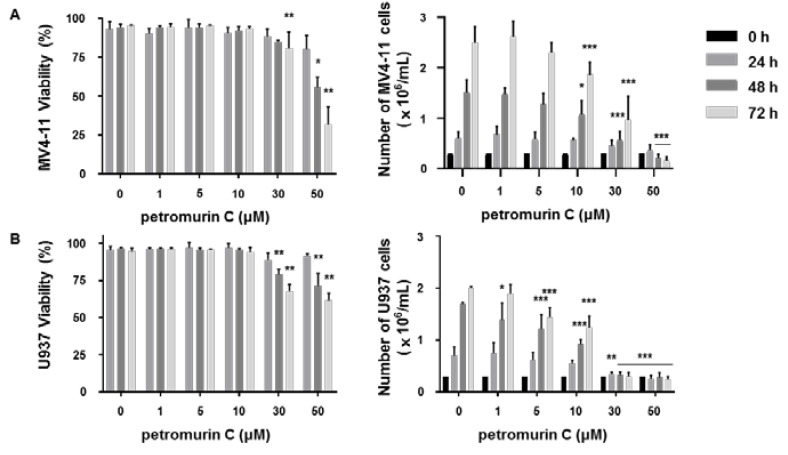

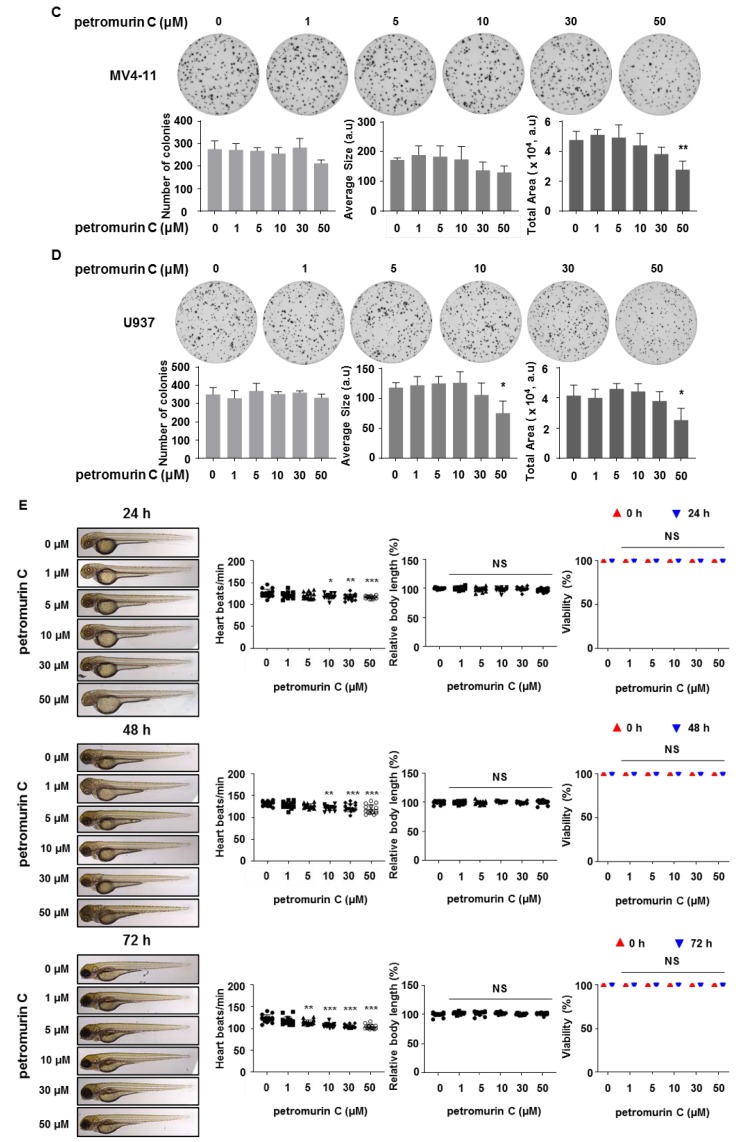
Anti-cancer effect of petromurin C against AML cells. Effect of petromurin C on the cell number and viability of (**A**) MV4-11 cells and (**B**) U937 cells after 24, 48, and 72 h of treatment. Colony formation assays for (**C**) MV4-11 cells and (**D**) U937 cells treated with petromurin C (0–50 μM). Images are representative of three independent experiments. (**E**) In vivo zebrafish toxicity assays with quantification of heart rate, body lengths, and viability at 24, 48, and 72 h. The data are representative of three independent experiments with five embryos for each condition. All histograms represent the mean ± SD of at least three independent experiments. a.u.: arbitrary units. * *p* ≤ 0.05, ** *p* ≤ 0.01, *** *p* ≤ 0.001 compared to untreated cells. Two-way ANOVA (cell viability and proliferation). Post hoc: Dunnett’s test. One-way ANOVA (colony formation assay). Post hoc: Dunnett’s test. One-way ANOVA (heart beats/min, relative body length). Post hoc: Sidak’s test. Two-way ANOVA (zebrafish viability). Post hoc: Sidak’s test.

FLT3-ITD-mutated MV4-11 and FLT3 wild-type (WT) U937 cells were treated with increasing concentrations of petromurin C for up to 72 h. Petromurin C reduced proliferation and viability of MV4-11 and U937 AML cell lines (Figure 2A,B) as shown by trypan blue staining assays. The concentrations that reduced 50% of viability and proliferation are shown in Table 2.

We confirmed our results by colony formation assays (CFA) that showed a significant decrease of total colony areas at 50 μM in MV4-11 cells and at 50 μM in U937 cells (Figure 2C,D). To evaluate the in vivo toxicity of petromurin C, heart rate, body length, and viability of zebrafish embryos were analyzed after exposure to increasing concentrations of petromurin C. Although a heart rate of zebrafish decreased in a dose-dependent manner, there were no significant changes of viability and morphology of the zebrafish (Figure 2E).

### 2.3. Petromurin C Induces Cell Death

**Figure 3:**
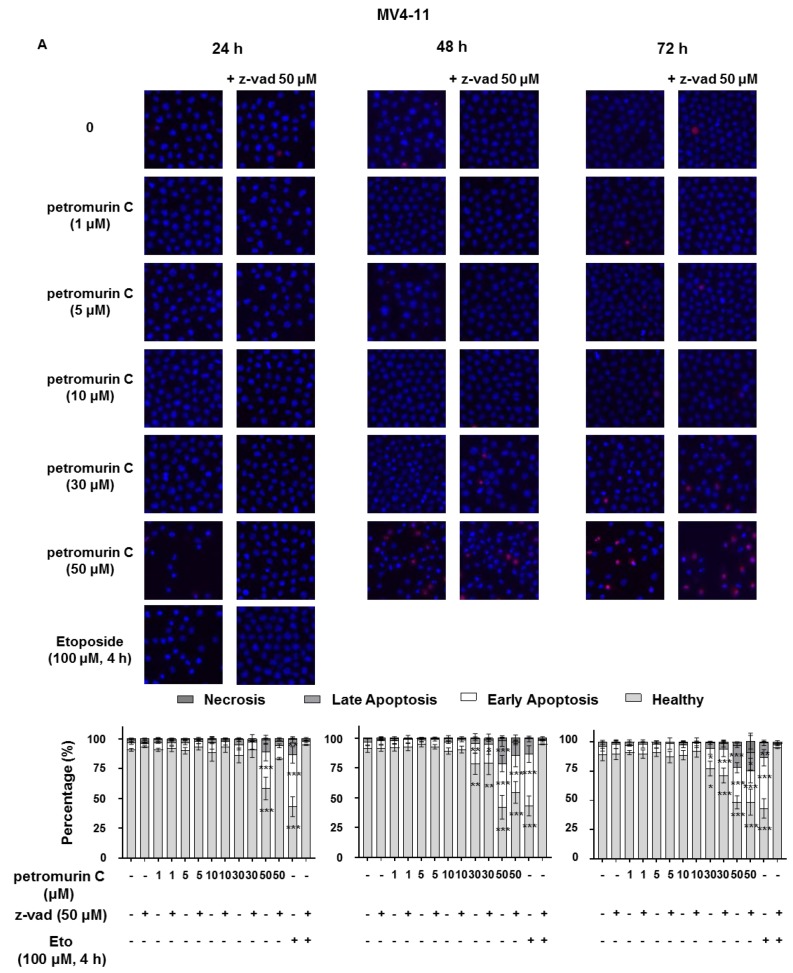

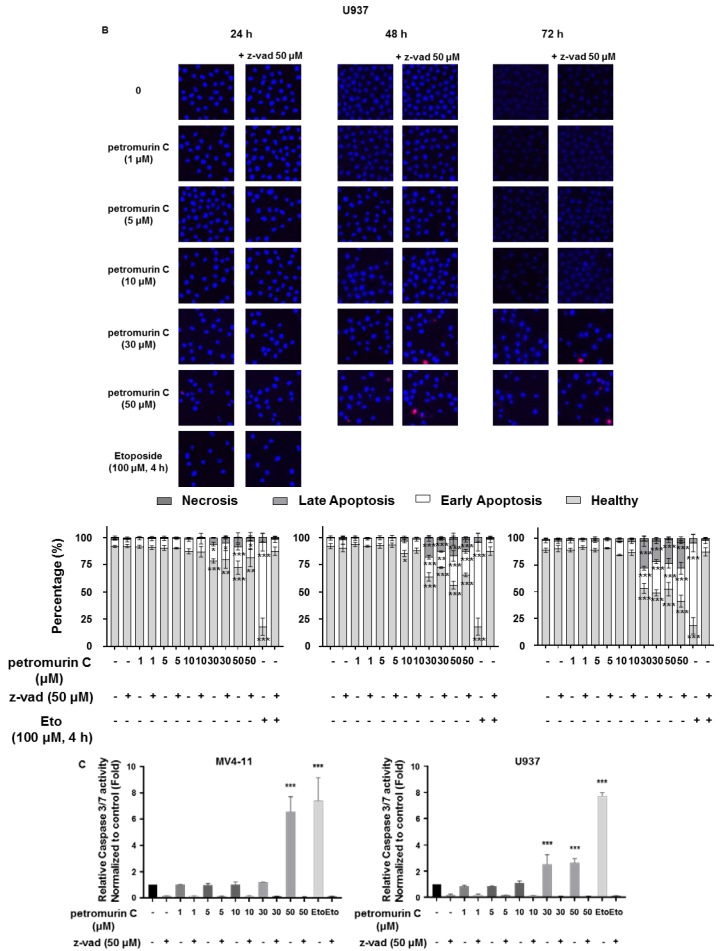
Caspase-dependent and Caspase-independent apoptosis induced by petromurin C in AML cell lines. Cell death induced by petromurin C in (**A**) MV4-11 cells and (**B**) U937 cells were identified by Hoechst/PI staining. (**C**) Caspase 3/7 activity of MV4-11 and U937 cells was assessed at 24 h. The cells were treated with petromurin C followed by a 1-h pretreatment with and without zVAD-FMK. Etoposide (Eto) was used as a positive control for apoptosis induction. All histograms represent the mean ± SD of at least three independent experiments. * *p* ≤ 0.05, ** *p* ≤ 0.01, *** *p* ≤ 0.001 compared to untreated cells. Two-way ANOVA (microscopy analysis). Post hoc: Dunnett’s test. One-way ANOVA (caspase 3/7 assay). Post hoc: Sidak’s test.

Type and characteristics of cell death induced by petromurin C were investigated by Hoechst/PI staining assays in AML cell lines. Nuclear condensation and fragmentation, which are characteristics of apoptotic cell death, were observed. Petromurin C significantly induced 41.7% of cell death at 50 μM after 24 h in MV4-11 cells, whereas lower concentrations did not affect viability at 24 h. zVAD-FMK (50 μM), which is a pan-caspase inhibitor, prevented apoptotic cell death in both cell lines at 24 h (Figure 3A,B). Similarly, petromurin C induced a 6.6-fold increase of caspase 3/7 activation at 50 μM in MV4-11 cells. We observed a 2.5-fold and 2.6-fold increase of caspase 3/7 activation at 30 and 50 μM in U937 cells. zVAD-FMK pretreatment inhibited caspase 3/7 activity in both cell lines at 24 h (Figure 3C). At later time points, zVAD-FMK pretreatment did not efficiently abrogate cell death in MV4-11 and U937 cell lines since 58.2% of cell death was induced by petromurin C at 50 μM in MV4-11 cells compared to 45.7% of cell death being induced by zVAD-FMK pretreatment at 48 h (Figure 3A,B), which allows us to hypothesize that other caspase-independent, cell death modalities are also induced by petromurin C, which is compatible with an irreversible mitochondrial commitment to death.

### 2.4. Petromurin C Activates the Mitochondrial Cell Death Pathway via Inhibition of Mcl-1 and Activation of Pro-Caspases 3/7 and 9

**Figure 4:**
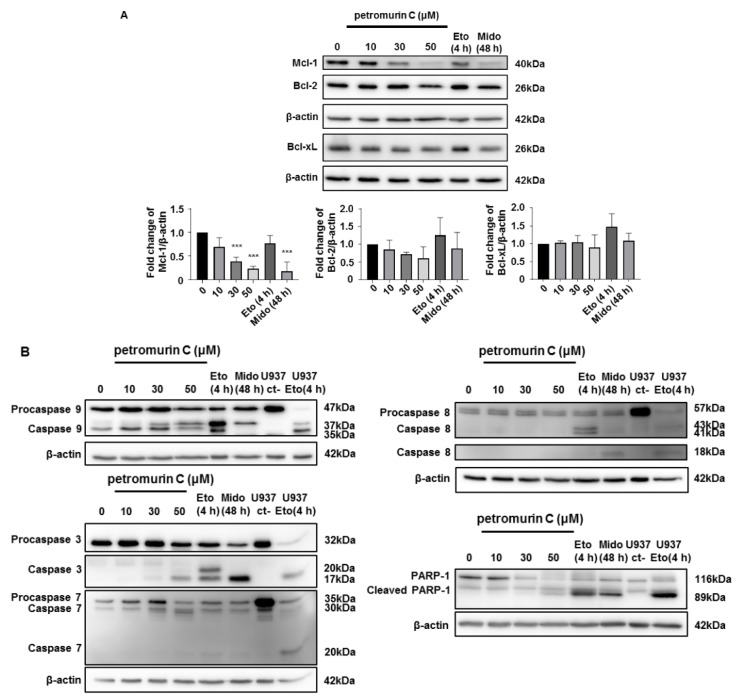
Expression levels of apoptotic and anti-apoptotic proteins treated with petromurin C. (**A**) Western blot analysis of Bcl-2 family proteins Mcl-1, Bcl-2, and Bcl-xL in MV4-11 cells treated with petromurin C (0, 10, 30, 50 μM) for 24 h (upper panel). Expression levels of the Bcl-2 family proteins were quantified (lower panel). (**B**) Western blot analyses of caspases 3, 7, 8, 9 and PARP-1 cleavage in MV4-11 cells. 100 μM of etoposide (Eto) and 330 nM of midostaurin (Mido) were used as positive controls for apoptosis induction. U937 cells treated with or without etoposide were utilized as a comparison of FLT3-wild type AML cell line. Blots are representative of three independent experiments. All histograms represent the mean ± SD of at least three independent experiments. *** *p* ≤ 0.001 compared to untreated cells. One-way ANOVA (Western blot). Post hoc: Dunnett’s test.

To study apoptotic cell death mechanisms induced by petromurin C, expression levels of apoptotic and anti-apoptotic proteins including Bcl-2, Bcl-xL (B-cell lymphoma-extra-large), and Mcl-1 (myeloid cell leukemia-1) were assessed in MV4-11 cells [13]. As shown in Figure 4A, Bcl-2 and Bcl-xL expression levels remained unchanged while levels of Mcl-1 were decreased by 61.4% and 77.1% at 30 and 50 µM, respectively, at 24 h in MV4-11 cells (Figure 4A).

Moreover, our results showed no cleavage of pro-caspase 8 at any concentration of petromurin C whereas pro-caspase 9 was cleaved dose-dependently. Cleavage of executioner pro-caspases 3 and 7 was detected at 50 µM of petromurin C (Figure 4B). The results from the caspase 3/7 activation assay in Figure 3C are in line with the results obtained by a Western blot.

To further validate the involvement of caspases, levels of cleaved PARP-1 (poly (ADP-ribose) polymerase-1) protein, which is a cellular substrate of caspases, were assessed after treatment with various concentrations of petromurin C [14]. The cleavage product of PARP-1 was detected at 89 kDa, which indicates a typical caspase activation, and is increased dose-dependently (Figure 4B). Clinically used chemotherapeutic agents etoposide (Etopophos) and midostaurin (Rydapt), a first generation FLT3 inhibitor, were used in all experiments as positive controls for apoptotic cell death induction.

### 2.5. Petromurin C Triggers Formation of Cytoplasmic Vesicles

**Figure 5:**
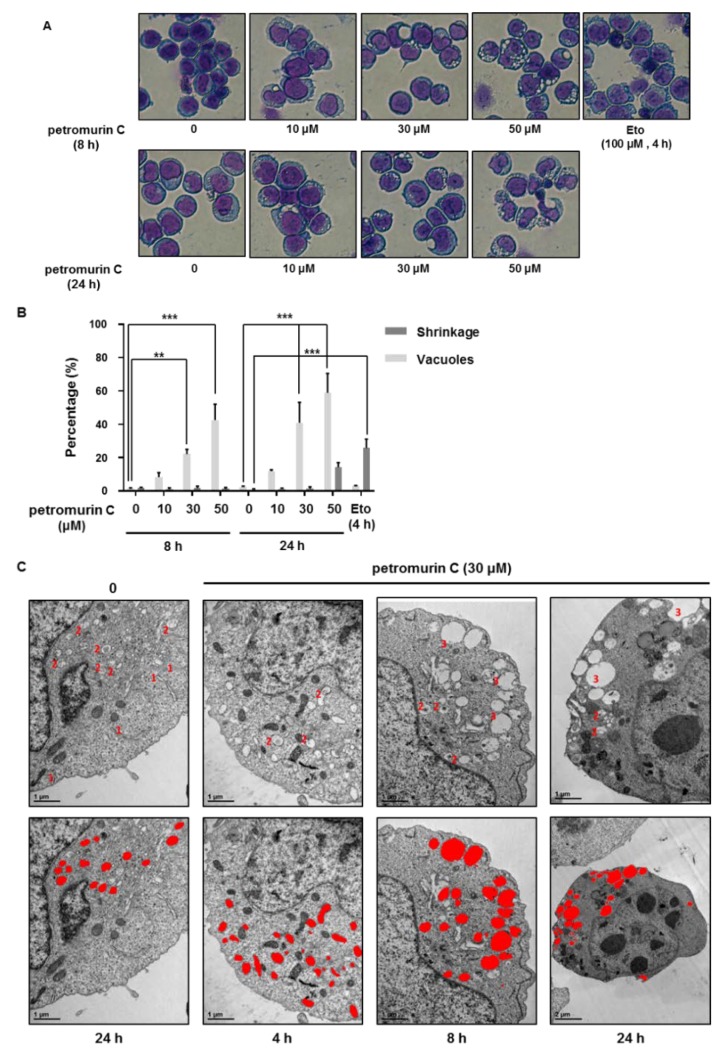
Morphological changes induced by petromurin C treatment. (**A**) Diff-Quik staining after treatment of MV4-11 cells at 8 and 24 h with 0, 10, 30, and 50 μM of petromurin C. Etoposide (Eto) was used as a positive control for apoptosis induction. (**B**) Quantification of cytoplasmic vesicles and shrinkages. (**C**) TEM images of MV4-11 cells treated with 0 or 30 μM of petromurin C for 4, 8, and 24 h at 25,000× magnification: (1) phagophores, (2) autophagolysosomes, (3) macro-autophagolysosomes (upper panel). Analysis of size differences of autophagolysosomes in TEM images of MV4-11 cells treated with 0 or 30 μM of petromurin C for 4, 8, and 24 h at 12,000 or 25,000× magnification (lower panel). ** *p* ≤ 0.01, *** *p* ≤ 0.001 compared to specified cells. Two-way ANOVA (Diff-Quik). Post hoc: Tukey’s test.

As we observed morphological changes in petromurin C-treated MV4-11 cells, we used Diff-Quik staining to visualize dose-dependent and time-dependent effects of petromurin C on MV4-11 cells. Extensive formation of cytoplasmic vesicles was observed starting at 10 μM, 8 h (Figure 5A). In addition, 8.3%, 22.1%, and 42.6% of cytoplasmic vesicles formed after 8 h at 10, 30, and 50 μM, respectively. At 24 h, 11.9%, 41.0%, and 58.9% of cytoplasmic vesicles formed at 10, 30, and 50 μM of petromurin C, respectively. In addition, 14.2% of pyknotic cells were observed at 50 μM after 24 h (Figure 5B).

Considering the extensive vesicle formation at 30 and 50 µM from 8 h and 41.7% of cell death observed in MV4-11 at 50 µM petromurin C (Figure 3A) after 24 h, we decided to investigate the cellular morphology by TEM (Transmission Electron Microscopy) at 30 µM petromurin C after 4, 8, and 24 h of treatment (Figure 5C). TEM images revealed that autophagolysosomes and “macro-autophagolysosomes” (see Material and Methods) were observed in petromurin C-treated MV4-11 cells. Areas of autophagolysosomes in 30 μM of petromurin C-treated MV4-11 cells at 24 h showed significant increases compared to the control, 4-h, and 8-h treated MV4-11 cells (Figure S1).

### 2.6. Petromurin C Triggers a Rapid, Protective Autophagic Flux

**Figure 6:**
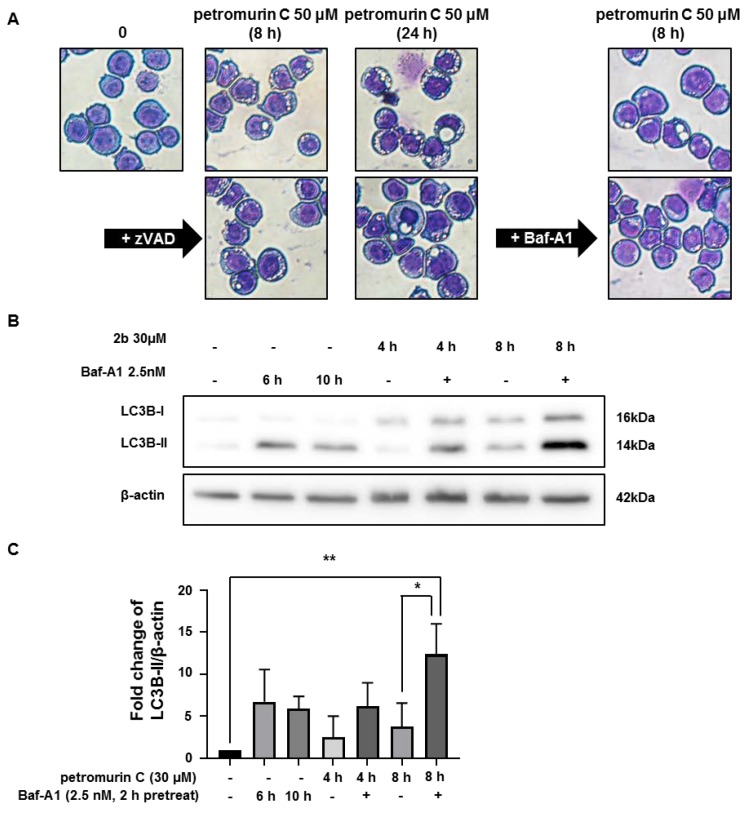

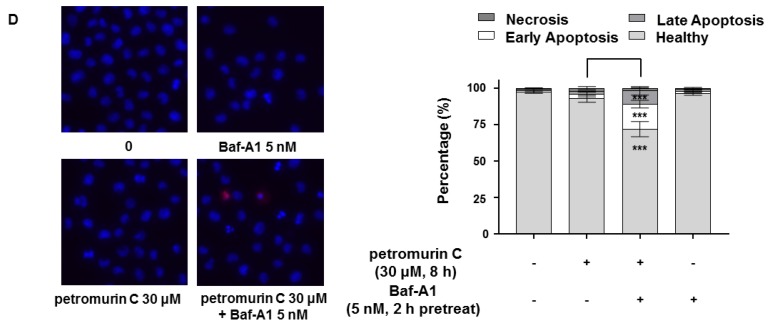
Induction of cytoplasmic vesicle formation and autophagy. (**A**) Diff-Quik staining of MV4-11 cells after 8 h of petromurin C treatment (50 μM) with or without zVAD-FMK (zVAD) or bafilomycin-A1 (Baf-A1) pretreatment. (**B**) Western blot analysis of the LC3B protein. β-actin was used as a loading control. (**C**) Quantification of LC3B protein bands through normalization by β-actin protein bands. (**D**) MV4-11 cells were pretreated with Baf-A1 for 2 h and, subsequently, treated with 50 μM of petromurin C for 8 h. Percentage of cell death was quantified after Hoechst/PI staining. All data represent the mean ± SD of at least three independent experiments. Baf-A1 was used as an autophagy inhibitor. * *p* ≤ 0.05, ** *p* ≤ 0.01, *** *p* ≤ 0.001 compared to specified cells. One-way ANOVA (Western blot). Post hoc: Tukey’s test. Two-way ANOVA (microscopy analysis). Post hoc: Tukey’s test.

In order to further assess the nature of the cytoplasmic vesicles observed in petromurin C-treated-MV4-11 cells, we co-treated our cells with caspase and autophagy inhibitors prior to Diff-Quik staining. Pan-caspase inhibitor, zVAD-FMK (50 µM, 1 h-pretreatment), did not prevent the formation of cytoplasmic vesicles at the highest concentration at 8 h. However, the autophagy inhibitor, bafilomycin A1 (Baf-A1, 5 nM, 2 h-pretreatment) significantly inhibited the formation of vesicles in the cytoplasm (Figure 6A). To validate the involvement of petromurin C in autophagy induction, we pretreated MV4-11 cells with 2.5 nM of Baf-A1 prior to treatment with petromurin C at 30 µM at different time points. The autophagy induction marker LC3B was quantified after immunoblotting. LC3B I-II conversion time-dependently increased by 6.2-fold and 12.4-fold for 6 and 10 h with a 2-h pretreatment of Baf-A1, respectively (Figure 6C), whereas p62 expression levels did not significantly change (Figure S2). To test which mode of autophagy petromurin C caused, we used Hoechst/PI staining of petromurin C-treated MV4-11 cells with or without Baf-A1 pretreatment. The results showed that 28.1% of cell death was observed in petromurin C-treated MV4-11 cells with a pretreatment of Baf-A1 compared to 7.3% of cell death in only petromurin C-treated MV4-11 cells (Figure 6D). According to the results, we concluded that Baf-A1 inhibited the formation of autophagy and significantly potentialized petromurin C-induced cell death at early time points.

### 2.7. Petromurin C Generates Mitochondrial Membrane Potential Loss and Mitochondrial Stress

**Figure 7:**
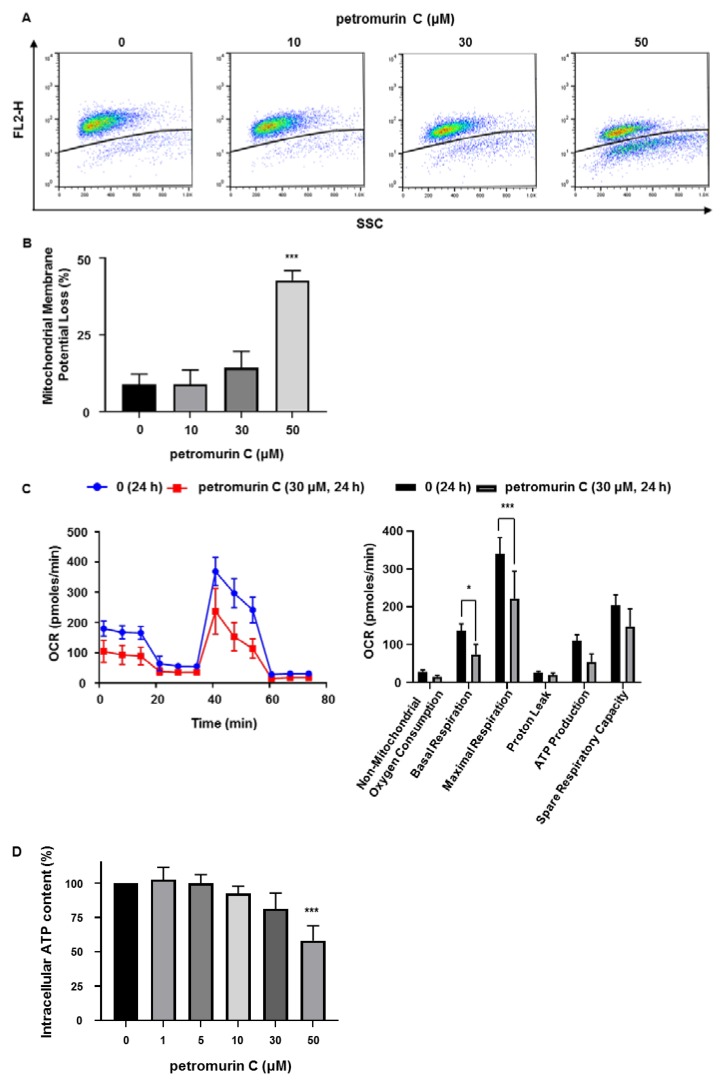
Effects of petromurin C on mitochondrial membrane potential (MMP) and stress. (**A**) MMP loss of petromurin C-treated MV4-11 after 24 h by MitoTracker Red CMXRos staining. (**B**) Quantification of the percentage of MMP loss. (**C**) Oxygen consumption rate (OCR) of MV4-11 cells treated with 30 μM of petromurin C for 24 h was measured by a Seahorse XFp analyzer. (**D**) Quantification graphs of non-mitochondrial oxygen consumption, basal respiration, maximal respiration, proton leak, ATP production, and spare respiration capacity. (**E**) Intracellular ATP levels of MV4-11 cells treated with different concentrations of petromurin C at 24 h. All data represent the mean ± SD of at least three independent experiments. * *p* ≤ 0.05, ** *p* ≤ 0.01, *** *p* ≤ 0.001 compared to untreated cells. One-way ANOVA (mitochondrial membrane potential loss), post hoc: Dunnett’s test. Two-way ANOVA (mito stress test), post hoc: Sidak’s test. One-way ANOVA (cell titer glo assay), post hoc: Dunnett’s test.

Since mitochondrial dysfunction is strongly related to the induction of apoptosis, we measured the percentage of mitochondrial potential loss at 24 h in MV4-11 cells by FACS after staining with MitoTracker Red CMXRos (Figure 7A). Our results showed a dose-dependent increase of mitochondrial membrane potential loss (42.7% at 50 μM) (Figure 7B). These results are in agreement with the increased activation of caspase 9 (Figure 4B).

Next, to investigate mitochondrial stress caused by petromurin C treatment, we evaluated the oxygen consumption rate (OCR) (Figure 7C). In response to petromurin C treatment, we observed decreases of non-mitochondrial oxygen consumption (1.9-fold), basal respiration (1.9-fold), maximal respiration (1.5-fold), proton leak (1.4-fold), ATP production (2.0-fold), and spare respiratory capacity (1.4-fold) (Figure 7D). The initial OCR level of petromurin C-treated MV4-11 cells was lower than that of control cells, which indicated a decline of total mitochondrial mass. Oligomycin, an ATP synthase inhibitor, notably reduced ATP-linked respiration in control MV4-11. Maximal respiration of both control and petromurin C-treated MV4-11 cells increased after injection of the mitochondrial uncoupler FCCP (Figure 7C). However, the maximal respiration rate decreased in petromurin C-treated MV4-11 cells by 45.4% when compared to control cells (Figure 7D). The intracellular ATP levels of cells treated with petromurin C decreased dose-dependently and significantly at 50 µM (Figure 7E). Altogether, these results are in line with the loss of mitochondrial membrane potential.

### 2.8. Synergistic Effects of a Combination of Petromurin C and Gilteritinib on Cell Death

**Figure 8:**
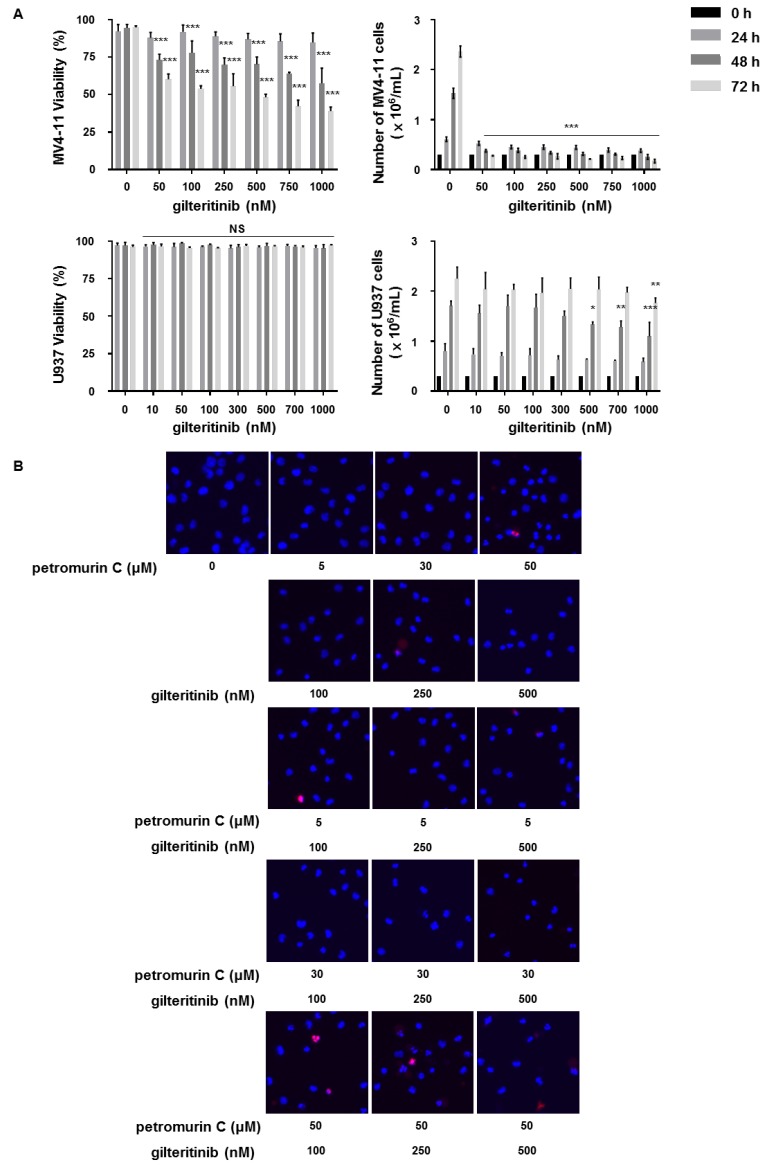

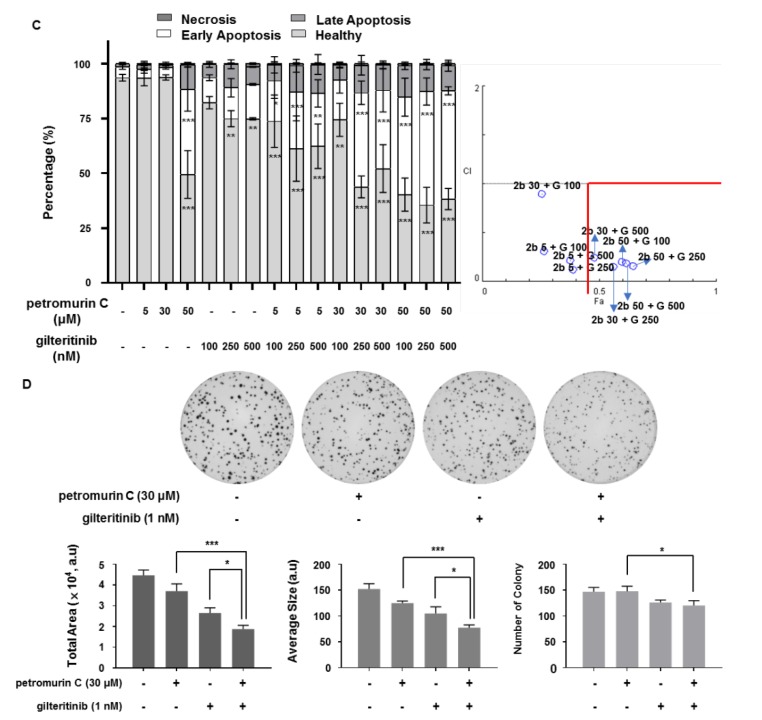
Synergistic effect of the combination of petromurin C and gilteritinib. (**A**) Effect of gilteritinib on cell number and viability of MV4-11 and U937 cells after 24, 48, and 72 h of treatment. (**B**) Hoechst/PI staining of a combination treatment of petromurin C and gilteritinib in MV4-11 cells. (**C**) Quantification of cell death modalities triggered by a combination of petromurin C and gilteritinib (left panel). An estimation plot of the combination treatment by Compusyn software with combination index (CI) values (*y*-axis) and fraction affected (fa, *x*-axis) (right panel). Petromurin C (2b) and gilteritinib (G) were used for the calculation. (**D**) Colony formation assay of a combination treatment of 30 μM of petromurin C and 1 nM of gilteritinib in MV4-11 cells. All data represent the mean ± SD of at least three independent experiments. * *p* ≤ 0.05, ** *p* ≤ 0.01, *** *p* ≤ 0.001 compared to untreated cells unless otherwise specified. Two-way ANOVA (cell viability, proliferation, microscopy analysis). Post hoc: Dunnett’s test. One-way ANOVA (colony formation assay). Post hoc: Tukey’s test.

Since gilteritinib (ASP2215), which is a dual inhibitor of FLT3/AXL, has been clinically studied in combination treatments, we attempted to combine it with petromurin C. Viability and proliferation assays with gilteritinib were first quantified in U937 and MV4-11 cells. Results showed that gilteritinib specifically targets FLT3-ITD-mutated MV4-11 cells while this compound has no effect on viability and proliferation of U937 cells expressing wild-type FLT3 (Figure 8A). In the next step, the cell death modality induced by gilteritinib was characterized and quantified by fluorescence microscopy after Hoechst/PI staining in MV4-11 cells. Since the pan-caspase inhibitor zVAD-FMK (50 μM) reduced the percentage of cell death induced by gilteritinib (Figure S3A,B), we concluded that caspase-dependent apoptosis was induced.

To assess the combinatorial effect of both compounds, we initially used suspension cultures and combined 5, 30, and 50 μM of petromurin C with 100, 250, and 500 nM of gilteritinib. The results obtained by Hoechst/PI staining after 24 h allowed us to calculate the combination index (CI) and Fraction affected values (Fa) according to Chou-Talalay (Figure 8B,C). Our results showed a synergistic effect of the combination of petromurin C at 30 μM and gilteritinib at 250 and 500 nM as well as petromurin C at 50 μM and gilteritinib at all three concentrations (Table 3). The combination of petromurin C at 30 μM and gilteritinib at 250 nM showed the strongest synergistic effect compared to both drugs used individually.

We then aimed to validate our data by using CFAs. As gilteritinib at 250 nM alone completely eradicated colony formation, concentrations of 0.5, 1, and 2 nM were tested alone and in combination with petromurin C (Figure S4A). Combination index (CI) values were calculated using average colony size values of 10, 30, and 50 μM of petromurin C with 0.5, 1, and 2 nM of gilteritinib (Figure S4B,C). CI values showed a synergistic effect, besides petromurin C (10 μM)/gilteritinib (0.5 nM) (Table 4). In addition, the combination of 30 μM of petromurin C and 1 nM of gilteritinib significantly reduced the total area and average size of MV4-11 colonies compared to treatments of petromurin C and gilteritinib alone at subtoxic concentrations (Figure 8D).

### 2.9. Investigation of Morphological Changes after Combination Treatments 

**Figure 9:**
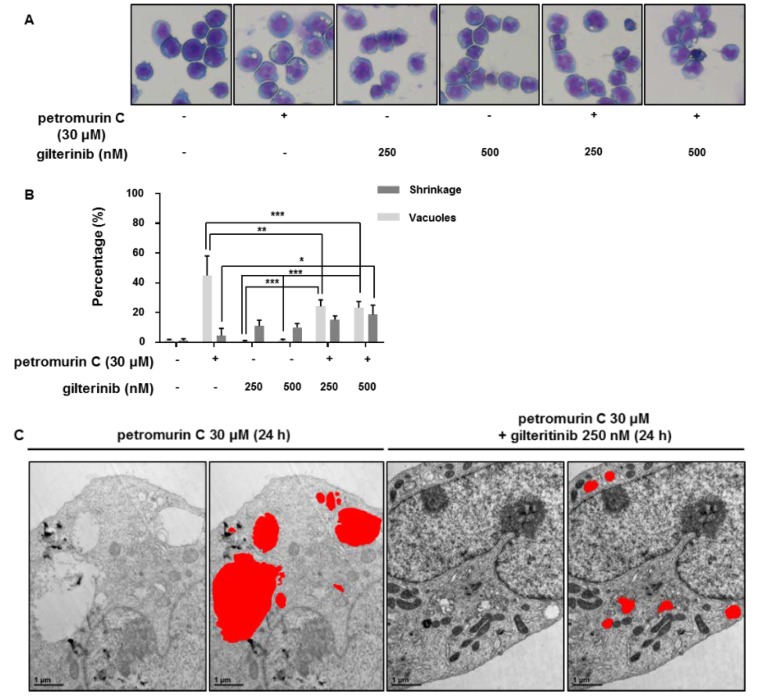
The combination treatment of petromurin C and gilteritinib triggered morphological changes in MV4-11 cells. (**A**) Diff-Quik staining of MV4-11 cells treated with a combination of petromurin C at 30 μM and gilteritinib at 250 nM at 24 h. (**B**) Quantification of Diff-Quik staining of MV4-11 cells treated with a combination of petromurin C at 30 μM and gilteritinib at 250 nM at 24 h. (**C**) Analysis of size differences of autophagolysosomes in transmission electron microscopy (TEM) images of MV4-11 cells treated with a combination of petromurin C at 30 μM and gilteritinib at 250 nM at 24 h at 25,000× magnification. * *p* ≤ 0.05, ** *p* ≤ 0.01, *** *p* ≤ 0.001 for the indicated comparisons. Two-way ANOVA (Diff-Quik). Post hoc: Tukey’s test.

Next, we used Diff-Quik staining (Figure 9A) and TEM images (Figure 9C) of MV4-11 cells treated with a combination of petromurin C and gilteritinib at 24 h to visualize morphology of the cells. Our results showed cell shrinkage (apoptotic volume decrease, AVD) and induction of vesicle-formation after combination treatment. Furthermore, 30 µM of petromurin C alone led to 44.9% of MV4-11 cells that formed vacuoles with 4.4% of cells undergoing AVD. On the other hand, 24.2% of vacuole-forming cells and 15.2% of AVD were observed after a combination treatment of 30 µM of petromurin C and 250 nM of gilteritinib. Additionally, 23.3% of vacuole-forming cells and 18.7% of AVD were observed after a combination of 30 µM of petromurin C and 500 nM of gilteritinib in MV4-11 cells (Figure 9B). We observed that a combination of petromurin C and gilteritinib induced a decrease of vacuole formation while it increased AVD of the cells.

Autophagolysosomes are a dominant feature in MV4-11 cells treated with both 30 µM of petromurin C or a combination of 30 µM of petromurin C and 250 nM of gilteritinib after 24 h (Figure 9C). The size and number of cells are smaller after the combination treatment when compared to petromurin C alone (Table 5). In addition, 42.2% of autophagolysosomes with an area bigger than 0.2 µm^2^ were found in 30 µM petromurin C-treated MV4-11 cells while only 12.0% in combination-treated cells. A smaller autophagolysosomal size could indicate an indirect feature of delayed auto-phagocytosis.

## 3. Discussion

The five-year overall survival rates for patients who are under 60 years old are 35%–40% with a median overall survival of one year [15]. FLT3-activating mutations are one of the most frequently found genetic aberrations in AML. Especially, FLT3-ITD mutations were associated with the worst prognosis. Efforts to improve outcomes for such AML patients were impeded by numerous obstacles including drug resistance and epigenetic modulations over the past 15 years [16]. After a long stagnation in the drug development process to treat leukemia, clinical options have been changed rapidly with drugs such as the first-generation multi-kinase inhibitor midostaurin, the dual FLT3/AXL inhibitor gilteritinib, the highly selective oral Bcl-2 inhibitor venetoclax, the isocitrate dehydrogenase (IDH)1 mutation inhibitor ivosidenib (which inhibits the formation of oncometabolite 2-hydroxyglutarate), the anti-CD33 antibody conjugated to the antibiotic calicheamicin (gemtuzumab ozogamicin) [17], and a first inhibitor of histone deacetylases (HDAC) classes I and II enzymes vorinostat (suberoylanilide hydroxamic acid, SAHA) [18]. Even though there have been improvements in targeted therapies in AML, patients who are initially responding to targeted therapies, eventually develop resistance due to various factors including epigenetic reprograming and genetic mutations [19]. Therefore, investigation of novel drugs to treat AML has been in a constant need.

Interest in marine natural products drug discovery increased as the number of anticancer drug development pipelines decreased [20]. Preclinical research with compounds derived from spongothymidine and spongouridine isolated from the Caribbean sponge *Tethya crypta* in 1950s progressively increased the approval rate of marine natural products [21]. However, unlike many terrestrial natural compounds such as taxol, obtained from the Western Pacific yew tree *Taxus brevifolia*, marine natural compounds remain less investigated [21]. In this case, we investigated the cytostatic and cytotoxic potential of petromurin C, a *bis*-indolyl benzenoid, isolated from the extract of the cultures of the marine-derived fungus *A. candidus* KUFA 0062, alone and in combination with gilteritinib in FLT3-positive AML. We first compared the cytostatic and cytotoxic potential of petromurin C in FLT3-mutated and FLT3-wild type AML cells. Petromurin C showed better efficacy in the FLT3-mutated cell line MV4-11 compared to FLT3-wild type U937 cells by showing a lower IC_50_ value and a significant decrease of the total colony area. We also confirmed the safety of petromurin C by in vivo toxicity assays on zebrafish. Since there were no effects on viability and morphology of the zebrafish, we considered petromurin C to be a safe drug to be studied since it also complied with Lipinski’s rules of five.

A previous study showed that petromurin C reduced the number of viable cells of various cancer cell lines including liver, lung, breast, and colorectal adenocarcinoma. Among these cancer cell lines, petromurin C was most effective in the colorectal adenocarcinoma cell line HT-29 [6]. Accordingly, fluorescent microscopy showed that petromurin C induced nuclear shrinkage and fragmentation, which are characteristics of the apoptotic cell death in AML cell lines. zVAD-FMK was able to prevent apoptotic cell death in both MV4-11 and U937 cell lines up to 24 h, hinting at caspase-dependent apoptotic cell death. However, in both cell lines, z-VAD-FMK did not prevent cell death at 48 and 72 h, which indicated caspase-independent cell death. There was also a finding that U937 cells treated with RS-F3, which was isolated from the marine sponge *Suberea clavate*, in the presence of the caspase inhibitor showed a shift to caspase-independent apoptotic-like cell death [5].

Further molecular mechanisms of petromurin C were studied by assessing levels of apoptotic and anti-apoptotic proteins. We showed that petromurin C reduced Mcl-1 levels. Other marine natural compounds such as marinopyrrole A [22] and RS-F3 [5] have been shown to reduce Mcl-1 levels as well. Furthermore, our study showed that petromurin C triggered a dose-dependent activation of caspase 9, which is followed by a cleavage of pro-caspases 3 and 7 in the MV4-11 cell line at 24 h. The involvement of activation of caspase 9 is known to catalyze the cleavage of pro-caspases 3 and 7 into their active forms, which have a role in cell demise during the intrinsic cell death pathway [8]. Our results are similar to those of the dihydroxyanthraquinone physcion, which is a secondary metabolite of the marine-derived fungus *Microsporum* sp. This induced apoptosis by downregulating anti-apoptotic proteins and activation of caspases 3 and 9 [23]. In addition to activation of caspase 9, mitochondrial outer membrane permeabilization (MOMP) is also responsible for cell death via intrinsic apoptosis [8]. Since members of the Bcl-2 family are known to antagonize MOMP, decreased protein levels of Mcl-1 in petromurin C-treated MV4-11 cells suggested a possibility of mitochondrial dysfunction. MOMP leads to dispersion of the mitochondrial transmembrane potential by the cytosolic release of apoptogenic factors including cytochrome c, which allows electron transport between complexes III and IV in the mitochondrial respiratory chain. Consequently, loss of mitochondrial transmembrane potential and cytochrome c release lead to respiratory impairment and decreased ATP production [8]. We observed increased mitochondrial membrane potential loss and decreased oxygen consumption rate after petromurin C treatment. Our results validated that petromurin C triggered cell death in MV4-11 cells via mitochondrial dysfunction and the intrinsic cell death pathway.

Autophagy is a process that is induced by cellular stress. Autophagy secretes cytoplasmic constituents and shows modulative activity in cancer [24]. Macroautophagy, which is the most studied type of autophagy, involves the activation of an autophagy receptor, LC3, required for the degradation of cellular contents. Crucial steps such as phagophore formation, elongation, and fusion with a lysosome lead to the formation of auto-phagolysosomes [25]. An increase of vacuoles in the cytoplasm was observed by Diff-Quik staining and TEM images after petromurin C treatment. TEM image analysis proved that the cytoplasmic vacuoles are auto-phagolysosomes, which are the fusion of LC3-decorated phagosomes with lysosomes [26]. We observed that pretreatment with an auto-phagosomal degradation inhibitor, Baf-A1, inhibited a formation of cytoplasmic vacuoles and increased conversion of LC3-I to LC3-II in MV4-11 cells after petromurin C treatment. Autophagy has a dual function. It can be an adaptive response in favor of cell survival against environmental stress whereas excessive autophagy induction can mediate cell death [7]. Hoechst/PI staining results of petromurin C-treated MV4-11 cells after Baf-A1 pretreatment supported the idea that autophagy plays a protective role in our case. Wan and colleagues also showed that coibamide A and apratoxin A, two cyclodepsipeptides isolated from cyanobacteria *Leptolyngbya* sp. and *Lyngbya* sp., respectively, induced caspase-dependent apoptosis preceded by autophagy [27]. Our study is consistent with their study since petromurin C-treated MV4-11 cells showed induction of autophagy at early time points followed by caspase-dependent and independent cell death at later times.

A previous study showed that petromurin C alone did not trigger antibacterial effects, while a synergistic effect was observed after combination with oxacillin against methicillin-resistant *Staphylococcus aureus* (MRSA) *S. aureus* 66/1, by using the disk diffusion method [6]. We then attempted a combination treatment of petromurin C. with gilteritinib, which is a specific FLT3 inhibitor that was clinically approved in November 2018 by FDA. Our results showed a synergistic effect of the combination of petromurin C and gilteritinib after quantification of nuclear condensation and cellular shrinkage. MV4-11 cells treated with the combination are more vulnerable to cell demise and show a decreased number and size of autophagolysosomes.

A combination treatment of petromurin C with gilteritinib revealed differential results in the 2D and 3D culture environment. 3D cell culture methods can create a more complex environment than 2D cell culture, mimicking an in vivo environment [28]. Tumor cells are known to be more resistant to drugs in 3D than in 2D cultures due to reduced access to compounds and pathophysiological differences such as hypoxia, which leads to activation of cell survival genes [29]. However, some studies showed that cells can also be more sensitive in 3D cultures compared to 2D cultures [30,31]. Melissaridou et al. showed head and neck squamous cell carcinoma cell line LK0902 was more sensitive to cetuximab, which is an epidermal growth factor receptor (EGFR) inhibitor in 3D conditions compared to 2D [30]. Adcock et al. also showed that the oral cancer cell line CAL27 was more sensitive to treatments by another EGFR inhibitor, erlotinib, under 3D conditions. Similarly, the semi-synthetic taxane docetaxel and the glycopeptide antibiotic bleomycin were more cytotoxic in 3D spheroids than in 2D cultures. The authors suggested that the increased sensitivity is associated with the relatively increased proliferation rate in 3D and increased levels of the expression of EGFR, which is the drug target, in 3D culture [31].

In 3D colony formation assays, lower concentrations of gilteritinib were required to achieve a synergistic effect with petromurin C when compared to a 2D culture. A potential explanation for this effect was recently shown by Adcock et al. since the differential proliferation rate of prostate cancer cell lines between 2D and 3D cultures was a dominant factor in determining the effect of selected anticancer drugs [31]. While we did not observe a specific growth advantage of MV4-11 when grown in 3D culture, we consider that the 3D environment favors cell growth in an autocrine way. Zheng et al. showed that the exogeneous FLT3 ligand triggered a four-fold increase of FLT3 autophosphorylation [32]. We suspect that gilteritinib most efficiently inhibits growth or reduces viability of MV4-11 cells growing under such conditions.

In addition, reduced levels of Mcl-1, loss of MMP, and mitochondrial dysfunction caused by petromurin C might contribute to the synergistic combination effect. Combination of gilteritinib and cytarabine (AraC), daunorubicin (DNR), idarubicin (IDR), or azacytidine (Aza) triggered inhibition of tumor growth and increased sensitivity to antitumor activity [11]. A combination treatment of gilteritinib with chemotherapy showed more efficacy compared to chemotherapy or gilteritinib alone in FLT3-ITD-mutated cell lines [11]. These results indicate that this combination treatment can be beneficial to patients who are not responding to gilteritinib when used alone.

## 4. Materials and Methods

### 4.1. Compound Isolation 

#### Sponge Collection and Compound Isolation

Petromurin C (compound **2b**) was provided from Interdisciplinary Centre of Marine and Environmental Research (CIIMAR), Terminal de Cruzeiros do Porto de Lexões, Av. General Norton de Matos s/n.

### 4.2. Biological Assays

#### 4.2.1. Chemicals

Etoposide (VP-16-213, E1383), midostaurin (M1213), bafilomycin (Baf-A1, #B1793) were purchased from Sigma-Aldrich (St. Louis, MO, USA). Caspase inhibitor 1 (zVAD-FMK, 627610) was purchased from Calbiochem (San Diego, CA, USA). Gilteritinib (ASP2215, S7754) was purchased from Selleckchem (Houston, TX, USA). All drugs were dissolved in DMSO.

#### 4.2.2. Cell Proliferation and Viability Assays

Acute Myeloid Leukemia cell lines U937 and MV4-11 were purchased from the Deutsche Sammlung von Mikroorganismen und Zellkulturen (DSMZ, Braunschweig, Germany). U937 cells were cultured in RPMI 1640 medium (Lonza, Walkersville, MD, USA) supplemented with 10% (*v*/*v*) fetal bovine serum (FBS), Biowest, Riverside, MO, USA) and 1% antibiotic–antimycotic (Lonza, Walkersville, MD, USA). MV4-11 cells were cultures in RPMI 1640 medium (Lonza, Walkersville, MD, USA) supplemented with 10% (*v*/*v*) FBS (Opti-Gold, GenDEPOT, Katy, Texas, TX, USA) and 1% antibiotic–antimycotic (Lonza, Basel, Belgium) at 37 °C in a humid atmosphere and 5% CO_2_.

The cell number and viability were measured by using the Trypan Blue exclusion assay (Lonza, Walkersville, MD, USA). The number of cells was counted in a Malassez cell counting chamber (Marienfeld, Lauda-Königshofen, Germany). 

#### 4.2.3. Colony Formation Assay 

A total of 1 × 10^3^ cells/mL were grown in semi-solid methylcellulose medium (StemCell Technologies Inc., Vancouver, Canada) supplemented with 10% FBS and indicated drug concentrations. After 10 days of culturing, 1 mg/mL of 3-(4,5-dimethylthiazol-2-yl)-2,5-diphenyl tetrazolium bromide (MTT) reagent (Sigma-Aldrich, St. Louis, MO, USA) was added to colonies. The colonies were detected by ImageQuant LAS 4000 mini system (GE Healthcare Life Science, Chicago, IL, USA) and quantified by ImageJ 1.52a software (U.S. National Institute of Health, Bethesda, MD, USA).

#### 4.2.4. Zebrafish Toxicity Assay

Zebrafish (*Danio rerio*) were obtained from the Zebrafish International Resource Center (ZIRC, University of Oregon, OR, USA), maintained according to SNU guidelines at 28.5 °C with 10 h dark/14 h light cycles. Zebrafish embryos were treated with 0.003% phenylthiourea (Sigma-Aldrich, St. Louis, MO, USA) to inhibit a formation of pigments 14 h before the toxicity assay. After dechorionation of the embryos 2 h before the assay, indicated concentrations of a drug were added to 24-well plates with the embryos. Heartbeat rate, viability, and development were assessed after 24, 48, and 72 h of drug treatment under a light microscopy (Zeiss, Oberkochen, Germany). The embryos were fixed onto a glass slide with 3% methylcellulose (Sigma-Aldrich, St. Louis, MO, USA). Pictures of the embryos were taken, and their body lengths were measured by ImageJ 1.52a software (U.S. National Institute of Health, Bethesda, MD, USA).

#### 4.2.5. Evaluation of Cell Death

The modes of cell death were determined and quantified by staining with 1 µg/mL Hoechst 33342 (Sigma-Aldrich, St. Louis, MO, USA) and 1 µg/mL propidium iodide (Sigma-Aldrich, St. Louis, MO, USA). Nuclear morphology analyses were observed by using fluorescence microscopy (Nikon Eclipse Ti-U, Tokyo, Japan).

Enzymatic activity of caspase 3/7 was assessed by the Caspase-Glo 3/7 Assay (Promega, Madison, WI, USA) following the manufacturer’s protocol. Intracellular ATP levels were measured by CellTiter-Glo Luminescent Cell Viability Assay (Promega, Madison, WI, USA) following the manufacturer’s protocol.

#### 4.2.6. Protein Extraction and Western Blots

Whole cell extracts were prepared using M-PER^®^ (ThermoFisher, Waltham, MA, USA) supplemented with 1× protease inhibitor cocktail (Complete EDTA-free, Roche, Basel, Switzerland), according to the manufacturer’s instructions. Proteins were resolved by sodium dodecyl sulfate polyacrylamide gel electrophoresis (SDS-PAGE) and transferred to polyvinylidene fluoride (PVDF) membranes (GE Healthcare Life Science, Chicago, IL, USA). Membranes were incubated with selected primary antibodies: anti-caspase 7 (9494S), anti-caspase 9 (9502S), anti-caspase 8 (9746), anti-Mcl-1 (4572S), anti-LC3B (2775), and anti-p62 (5114) from Cell Signaling (Danvers, MA, USA), anti-PARP-1 (C2-10; sc-53643) from Santa Cruz Biotechnology (Dallas, TX, USA), anti-Bcl-xL (610212) from BD Pharmingen (San Jose, CA, USA), and anti-β-actin (5441) from Sigma Aldrich (St. Louis, MO, USA). 

Luminescence signals were detected with the enhanced luminol-based chemiluminescent (ECL) Plus Western Blotting Detection Systems (GE Healthcare Life Science, Chicago, IL, USA). Bands were acquired by using an Amersham Imager 600 (GE Healthcare Life Science, Chicago, IL, USA) and quantified by ImageJ 1.52a software (U.S. National Institute of Health, Bethesda, MD, USA).

#### 4.2.7. Morphological Analysis

The cells were washed in 1× PBS and then spun onto glass slides for 5 min at 500 rpm using a Cytopad with caps (Elitech biomedical systems, Puteaux, France). The cells were fixed on the glass slides and stained with the Diff-Quik staining kit (Sysmex, Kobe, Japan), according to the manufacturer’s procedure. Images were acquired using a microscope (Nikon Eclipse Ti-U, Tokyo, Japan). A total of 100 cells were counted in one area. Three independent areas were counted for each set of three independent experiments. 

#### 4.2.8. Transmission Electron Microscopy (TEM) 

A total of 5 × 10^6^ cells were pelleted and fixed in 2.5% glutaraldehyde (Electron Microscopy Sciences, Hatfield, PA, USA), which was diluted in 0.1 M sodium cacodylate buffer, pH 7.2 (Electron Microscopy Sciences, Hatfield, PA, USA) for overnight. The cells were rinsed with sodium cacodylate buffer twice and then further fixed in 2% osmium tetroxide for 2 h at room temperature. Samples were washed with distilled water and then stained with 0.5% uranyl acetate at 4 °C overnight. After 24 h, samples were dehydrated through a graded series of ethanol solutions to water followed by propylene oxide, and then infiltrated in 1:1 propylene oxide/Spurr’s resin. Samples were kept overnight embedded in Spurr’s resin, mounted in molds, and left to polymerize in an oven at 56 °C for 48 h. Ultrathin sections (70–90 nm) were obtained with ultramicrotome, EM UC7 (Leica, Wetzlar, Germany). Sections were stained with uranyl acetate and lead citrate and, subsequently, examined with a JEM1010 transmission electron microscope (JEOL, Tokyo, Japan). All auto-phagolysosmes identifiable at the selected magnifications (12,000× and 25,000×) were segmented (red overlay in Figure 5C and Figure 9C) by Adobe Photoshop (San Jose, CA, USA) and measured by using FIJI software [33]. An area bigger than 0.2 µm^2^ was chosen to distinguish “macro-auto-phagolysosomes” (Table 5).

#### 4.2.9. Analysis of the Mitochondrial Membrane Potential

Cells were incubated at 37 °C for 30 min with 50 nM Mito-Tracker Red CMXRosRed (Molecular Probes, Invitrogen, Grand Island, NY, USA) and then analyzed by flow cytometry. Data were recorded statistically (10,000 events/sample) using the CellQuest Pro software. Data were analyzed using the Flow-Jo 8.8.7 software and results were expressed as mean fluorescence intensity (MFI).

#### 4.2.10. Determination of the Oxygen Consumption Rate

The oxygen consumption rate (OCR) was measured using a Seahorse XFp cell mito stress assay (#103010-100, Agilent, Santa Clara, CA, USA) ran on a Seahorse XFp analyzer (Agilent, Santa Clara, CA, USA), according to the manufacturer’s instructions. Cells were seeded at 100,000 cells per well and treated with petromurin C for 24 h in 175 μL medium. Before measurements, plates were equilibrated in a CO_2_-free incubator at 37 °C for 1 h. Analysis was performed using 1.5 µM oligomycin, 0.5 µM carbonyl cyanide-4-(trifluoromethoxy)phenylhydrazone (FCCP), and 1 µM rotenone/antimycin A, as indicated. Data were analyzed using the Seahorse XF Cell Mito stress rest report generator software (Agilent, Santa Clara, CA, USA).

#### 4.2.11. In Silico Drug-Likeness Properties

In silico drug-likeness properties according to Lipinski’s ‘rule-of-five’ and other parameters for drug-likeness and oral bioavailability were evaluated by using the SCFBio website (www.scfbio-iitd.res.in/).

#### 4.2.12. Statistical Analysis

GraphPad Prism 8.1.2 software (La Jolla, CA, USA) was utilized to statistically analyze data by one-way or two-way ANOVA. Data with *p*-values < 0.05 were considered as significant and represented by the following legend: * *p* ≤ 0.05, ** *p* ≤ 0.01, *** *p* ≤ 0.001. Data were expressed as the mean ± SD of at least three independent experiments. Combination index (CI) was calculated by using Compusyn Software (ComboSyn, Inc., Paramus, NJ, USA), following the instruction by Chou and Talalay. CI values < 1 and Fa values > 0.45 indicate synergism. Bar and whisker plots (Figure S1) were compared by a Kruskal-Wallis test followed by a Conover post-test further adjusted by the Benjamini-Hochberg false discovery rate (FDR)method (www.astatsa.com).

## 5. Conclusions

We showed that petromurin C induces an anticancer effect against AML cell lines. Our data indicate an important role of Mcl-1 and caspase 9 in the anticancer mechanisms of the drug. Furthermore, we revealed that loss of MMP and reduction of OCR are related to mitochondrial dysfunction, which eventually leads to cell demise. We also detected an involvement of autophagy early points as a protective mode after the drug treatment. Lastly, we suggested potential synergistic effects with the FLT3 inhibitor, gilteritinib, in eradication of an FLT3-ITD-mutated cell.

## Figures and Tables

**Table 1:** In silico prediction for the drug-likeness of petromurin C calculated and interpreted based on Lipinski’s rule of five [12].

Drug-Likeness	Petromurin C
Mass (g/mol)	444.49
Log*P*	5.26
Hydrogen bond donors	3
Hydrogen bond acceptors	7

**Table 2:** Inhibition of viability (IC_50_) and growth (GI_50_) of acute myeloid leukemia (AML) cell lines after 24, 48, and 72 h of treatment with petromurin C at increasing concentrations.

Cell Line	24 h		48 h		72 h	
	IC_50_ (µM)	GI_50_ (µM)	IC_50_ (µM)	GI_50_ (µM)	IC_50_ (µM)	GI_50_ (µM)
MV4-11	>50	>50	>50	18.56 ± 3.61	43.14 ± 2.88	19.28 ± 3.66
U937	>50	31.68 ± 6.83	>50	10.15 ± 3.42	>50	11.55 ± 2.34

**Table 3:** Calculation of the combination index values of petromurin C and gilteritinib in a suspension culture by Compusyn software.

Petromurin C (µM)	Gilteritinib (nM)	Effect (Fa)	CI
5	100	0.26	0.31
5	250	0.39	0.12
5	500	0.38	0.22
30	100	0.26	0.90
30	250	0.56	0.15
30	500	0.48	0.24
50	100	0.60	0.20
50	250	0.65	0.15
50	500	0.62	0.18

**Table 4:** Calculation of the combination index values of petromurin C and gilteritinib in colony formation assays with normalized average size values by Compusyn software.

Petromurin C (µM)	Gilteritinib (nM)	Effect (Fa)	CI
10	0.5	0.42	0.59
10	1.0	0.52	0.81
10	2.0	0.84	0.55
30	0.5	0.59	0.43
30	1.0	0.65	0.61
30	2.0	0.85	0.54
50	0.5	0.60	0.48
50	1.0	0.69	0.58
50	2.0	0.89	0.43

**Table 5:** Comparison for the number of vacuoles that have an area bigger or smaller than 0.2 μm^2^ and percentages of vacuoles with an area bigger than 0.2 μm^2^.

Vacuole Size/Area (µm^2^)	Petromurin C (30 µM)	Petromurin C (30 µM) and Gilteritinib (250 nM)
Area > 0.2 µm^2^	27	22
Area < 0.2 µm^2^	37	154
All	64	175
Area > 0.2 µm^2^ (%)	42.2	12.0

(*p* < 0.00001), Fisher exact test.

## Supplementary Materials

The following are available online at https://www.mdpi.com/1660-3397/18/1/57/s1. Figure S1: Box and whisker plots of areas of auto-phagolysosomes in 30 μM of petromurin C treated-MV4-11 cells after 4, 8, and 24 h. Figure S2: Induction of an active autophagic flux by petromurin C with or without Baf-A1 pretreatments. Figure S3: Nuclear morphology of caspase-dependent apoptosis induced by a single treatment of gilteritinib in MV4-11 cells. Figure S4: Effect of combination treatments of petromurin C and gilteritinib in the MV4-11 cell line.

## Abbreviations

AML: Acute myeloid leukemia. AraC: Cytarabine. ATP: Adenosine triphosphate. AVD: Apoptotic volume decrease. AXL: TAM family of receptor tyrosine kinases. Baf-A1: Bafilomycin A1. Bcl-2: B-cell leukemia/lymphoma 2. Bcl-xL: B-cell lymphoma extra-large. BSA: Bovine serum albumin. CFA: Colony formation assay. DMSO: Dimethyl sulfoxide. DNR: Daunorubicin. DSZM: Deutsche Sammlung von Mikroorganismen und Zellkulturen. ECL: Enhanced luminol-based chemiluminescent. EGFR: Epidermal growth factor receptor. FACS: Fluorescence-activated cell sorting. FCCP: Carbonyl cyanide-4 (trifluoromethoxy) phenylhydrazone. FBS: Fetal bovine serum. FDA: Food and drug administration. FDR: False discovery rate. FLT3: FMS-like tyrosine kinase 3. GI: Growth inhibition. HDAC: Histone deacetylases. HNSCC: Head and neck squamous cell carcinoma. HPF: Hours post fertilization. HSC: Hematopoietic stem cells. IC: Inhibitory concentration. IDH: Isocitrate dehydrogenase. IDR: Idarubicin. ITD: Internal tandem duplication. LC3B: Microtubule associated protein light chain 3B. Mcl-1: Myeloid cell leukemia-1. MMP: Mitochondrial membrane potential. MPER: Mammalian protein extraction reagent. MTT: 3-(4,5-dimethylthiazol-2-yl)-2,5-diphenyl tetrazolium bromide. OCR: Oxygen consumption rate. PARP-1: Poly (ADP-ribose) polymerase. PBS: Phosphate buffered saline. PI: Propidium iodide. PTU: Phenylthiourea. RTK: Receptor tyrosine kinase. PVDF: Polyvinylidene fluoride. RPMI: Roswell park memorial institute. SAHA: Suberoylanilide hydroxamic acid. SD: Standard deviation. SDS-PAGE: Sodium dodecyl sulfate polyacrylamide gel electrophoresis. SQSTM1: sequestosome-1. TAM: Tyro3, Axl, and Mertk homologous type I receptor tyrosine kinases. TEM: Transmission electron microscopy. WT: Wild type. Z-VAD-FMK: carbobenzoxy-valyl-alanyl-aspartyl-[O-methyl]-fluoromethylketone.
