# Supplementary material for: Petromurin C Induces Protective Autophagy and Apoptosis in FLT3-ITD-Positive AML: Synergy with Gilteritinib
Source: Mar Drugs. 2020 Jan 16;18(1):57. doi: 10.3390/md18010057 (PMC7024157; doi:10.3390/md18010057)
Supplement: Supplementary file 1 [file marinedrugs-18-00057-s001.pdf]

SUPPLEMENTARY MATERIAL

Petromurin C induces protective autophagy and apoptosis in FLT3-ITD-positive AML: synergy with gilteritinib

You Na Ha et al.

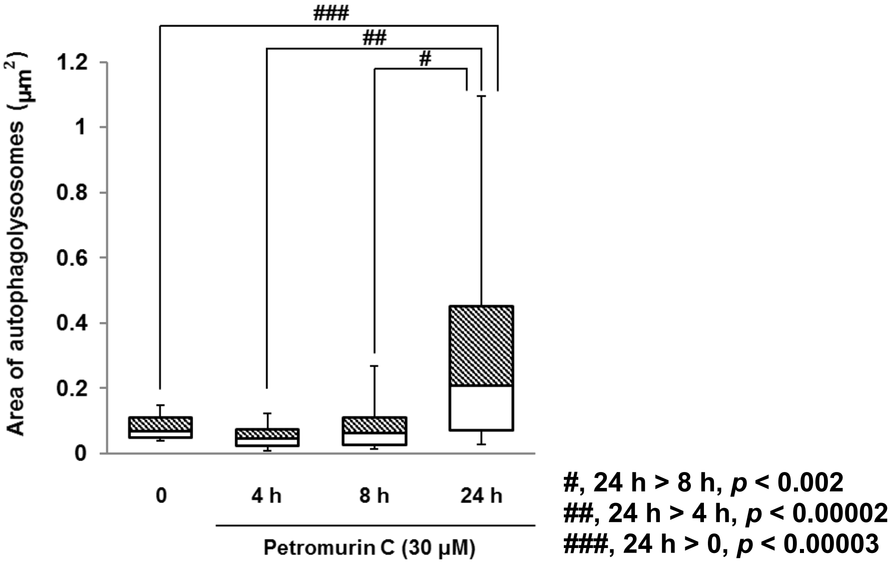

Supplementary Figure 1. Box and whisker plots of areas of autophagolysosomes in 30 μM of petromurin C treated-MV4-11 cells after 4, 8, and 24 h. Box and whisker plots: white box = lower interquartile range, hatched pattern box = upper interquartile range, whiskers = 10–90 percentile. The area of autophagolysosomes at 24 h is significantly bigger compared to that at 8 h with a p value smaller than 0.002. The area of autophagolysosomes at 24 h is significantly bigger compared to that at 4 h with a p value smaller than 0.00002. The area of autophagolysosomes at 24 h is significantly bigger compared to control with a p value smaller than 0.00003. Kruskal-Wallis test; post-hoc: Dunn's, further adjusted by the Benjamini-Hochberg FDR method.

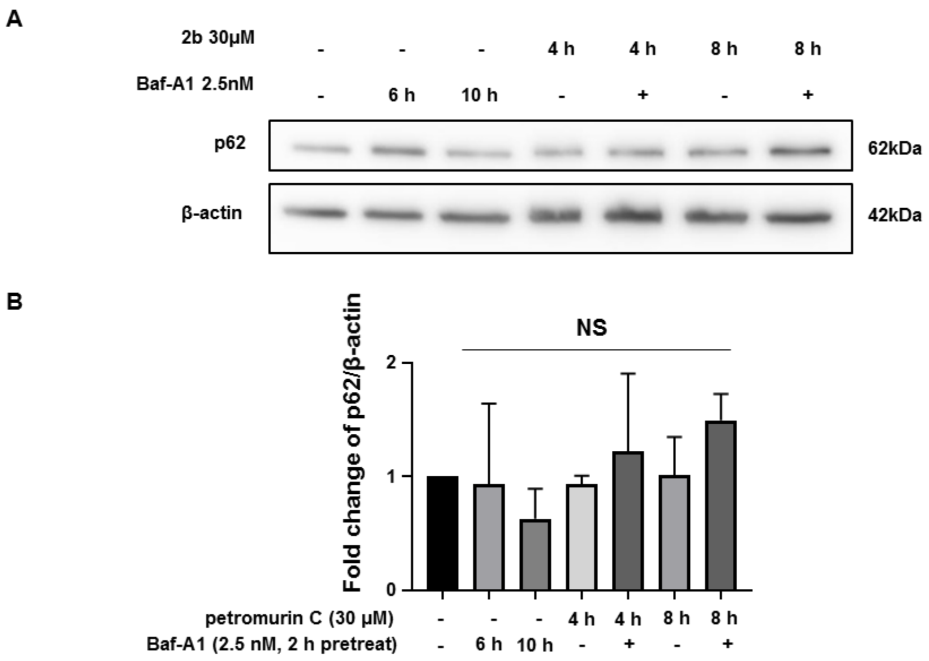

Supplementary Figure 2. Induction of active autophagic flux by petromurin C with or without Baf-A1 pretreatments. (A) Western blot analysis of p62. β-actin was used as a loading control. (B) Quantification of p62 protein bands through normalization by β-actin protein bands. NS compared to untreated cells. Two-way ANOVA (microscopy analysis); post hoc: Tukey's test.

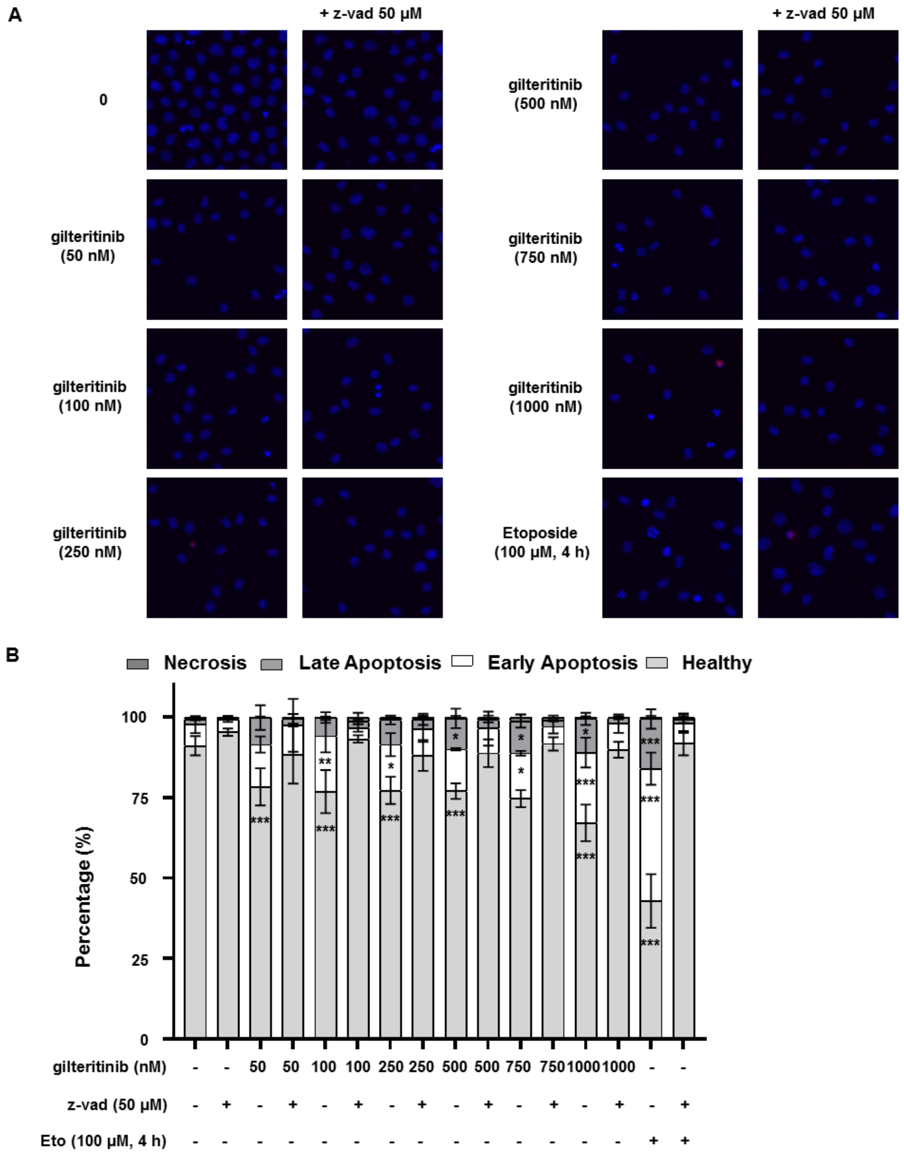

Supplementary Figure 3. Nuclear morphology of caspase-dependent apoptosis induced by a single treatment of gilteritinib in MV4-11 cells. (A) Effect of gilteritinib in MV4-11 cells after 24 h. (B) Quantification of apoptotic cell by nuclear morphology of gilteritinib after 24 h. All data represent the mean ± SD of at least 3 independent experiments. **p* ≤ 0.05, ***p* ≤ 0.01, ****p* ≤ 0.001 compared to untreated cells. Two-way ANOVA (microscopy analysis); post hoc: Tukey's test.

A

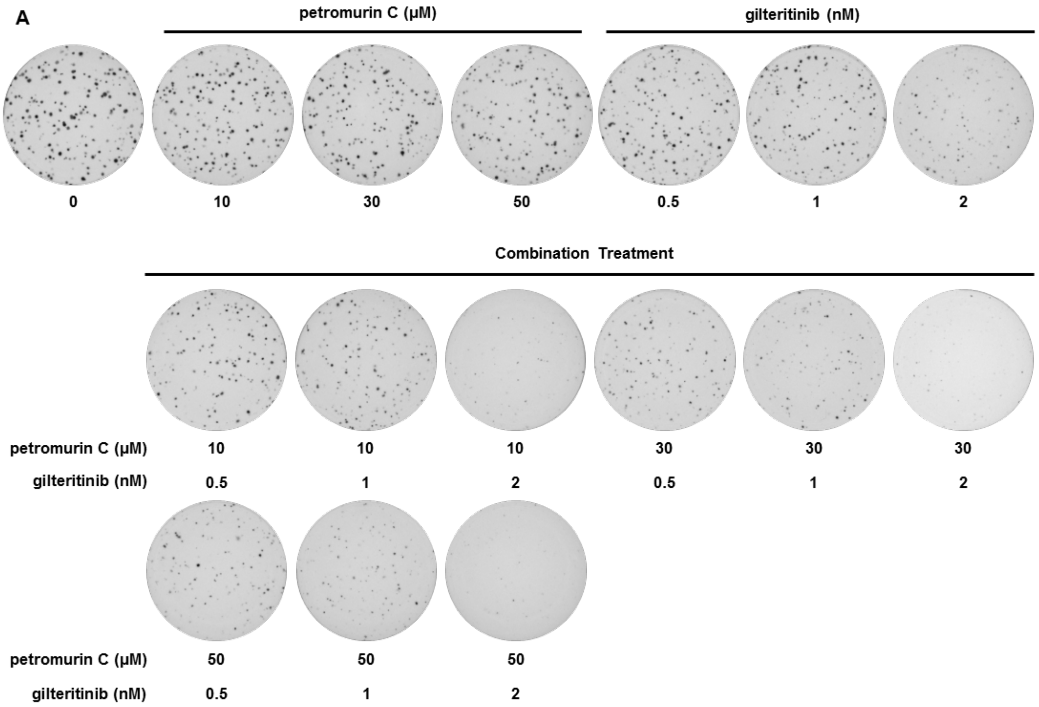
petromurin C (μM)
gilteritinib (nM)
0
10
30
50
0.5
1
2
Combination Treatment
petromurin C (μM) 10 10 10 30 30 30
gilteritinib (nM) 0.5 1 2 0.5 1 2
petromurin C (μM) 50 50 50
gilteritinib (nM) 0.5 1 2

B

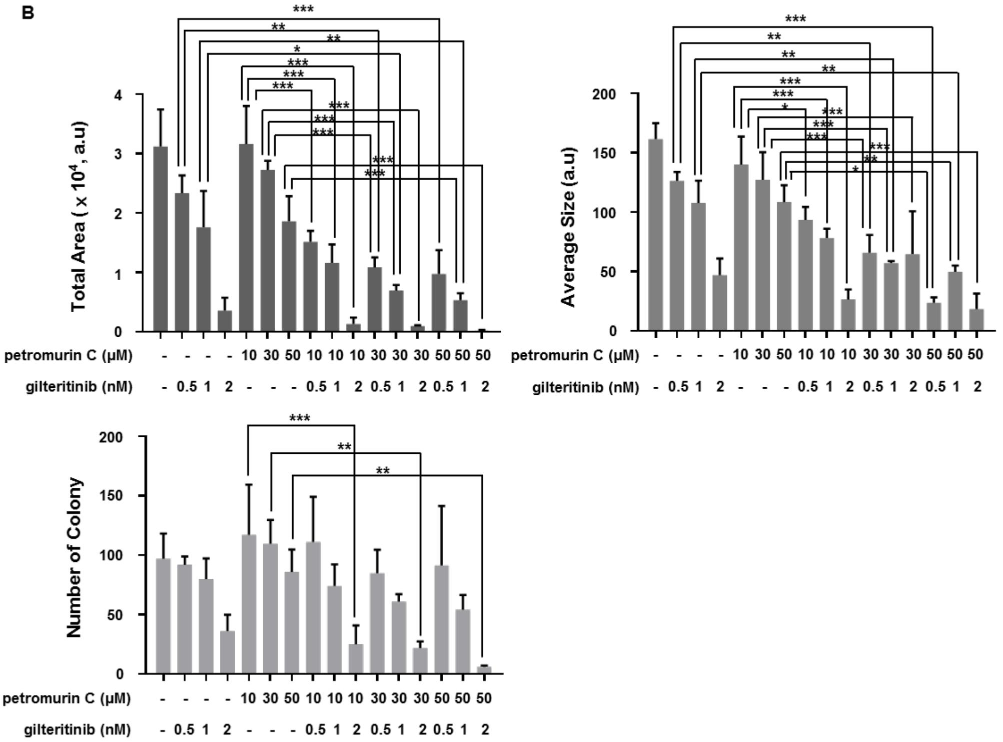
Total Area (x 10^4, a.u)
Average Size (a.u)
Number of Colony
petromurin C (μM) - - - - 10 30 50 10 10 10 30 30 30 50 50 50
gilteritinib (nM) - 0.5 1 2 - - - 0.5 1 2 0.5 1 2 0.5 1 2

C

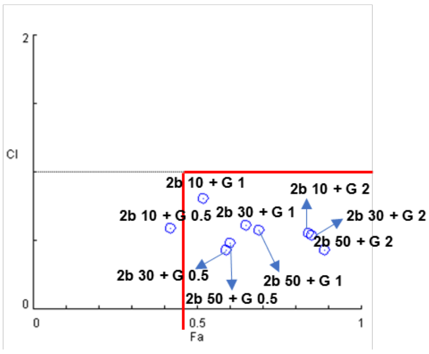
CI
Fa
2b 10 + G 1
2b 10 + G 2
2b 10 + G 0.5
2b 30 + G 1
2b 30 + G 2
2b 50 + G 2
2b 30 + G 0.5
2b 50 + G 1
2b 50 + G 0.5

Supplementary Figure 4. Effect of combination treatments of petromurin C and gilteritinib in MV4-11 cell line. (A) Colony formation assays for MV4-11 cells treated with the indicated concentrations of petromurin C. Images are representative of three independent experiments. (B) The quantification of the corresponding colony formation assays. (C) An estimation plot of the combination treatment by normalized average size from colony formation assays using Compusyn software with combination index (CI) values (y-axis) and fraction affected (fa, x-axis). Indicated concentrations of petromurin C (2b) and gilteritinib (G) were used for the calculation. All data represent the mean ± SD of at least 3 independent experiments. a.u.: arbitrary units. *$p \leq 0.05$, **$p \leq 0.01$, ***$p \leq 0.001$ compared for the indicated comparisons. One-way ANOVA (colony formation assay); post hoc: Sidak's test.
